# HLA molecules and nasal carriage of *Staphylococcus aureus *isolated from dialysis and kidney transplant patients at a hospital in Southern Brazil

**DOI:** 10.1186/1756-0500-5-90

**Published:** 2012-02-09

**Authors:** Luciana Borges Giarola, Rosiane Ribeiro dos Santos, João Bedendo, Waldir Veríssimo da Silva Júnior, Sueli Donizete Borelli

**Affiliations:** 1 Ingá/UNINGÁ-Maringá Nursing College, Universidade Estadual de Maringá (UEM), Maringá, Brazil; 2 Health Sciences Center, UEM, Maringá, Brazil; 3 Department of Nursing, UEM, Maringá, Brazil; 4 Department of Statistics, UEM, Maringá, Brazil; 5 Department of Basic Health Sciences, UEM, Maringá, Brazil; 6 Universidade Estadual de Maringá (UEM), Av. Colombo, 5790, Zona 07, Maringá, Paraná, Brazil 87020-900

## Abstract

**Background:**

Healthy individuals can host *Staphylococcus aureus *in the nasopharynx, body surface and vagina. Most invasive infections by this bacterium are endogenous, caused by strains spread from the nasopharynx of carriers. *S. aureus *is a pathogen involved in the etiology of hospital- and community-acquired infections. Transplant and dialysis patients are at risk of colonization or infection by multi-resistant *S. aureus*. Infection is directly linked to individual immunity, and the major histocompatibility complex (MHC) plays a crucial role in determining susceptibility to diseases. Different MHC specificities have been shown to be more frequent in individuals suffering from certain diseases. This study aimed to investigate the association between HLA class I (HLA-A and -B) and class II (HLA-DRB1) molecules and nasal carriage of *S. aureus *in dialysis and kidney transplant patients at a hospital in Southern Brazil.

**Results:**

The sample consisted of 70 dialysis and 46 kidney transplant patients, totaling 116 patients. All subjects were typed for HLA molecules using LABType^® ^SSO (One Lambda). Nasal swab samples of *S. aureus *were isolated from the nasal cavity (both nostrils) of patients undergoing dialysis or kidney transplantation.

In renal dialysis patients, HLA-A*02 was the most frequent allele in both carriers (25.5%) and non-carriers (21.2%) of *S. aureus*. Allele A*68 was not observed in the carrier group, but the allele was observed six times in the non-carrier group (*p *= 0.0097). Regarding HLA-B and HLA-DRB1, no allele was shown to be involved in protection against or susceptibility to carriage of *S. aureus*. In kidney transplant patients, allele A*03 was more frequent in the non-carrier (20.83%) than in the carrier (5.88%) group (*p *= 0.0486). HLA-B*15 was present in carriers (5.88%) and non-carriers (25%) (*p *= 0.0179). Regarding class II alleles, DRB1*03 appeared to be related to susceptibility to carriage of *S. aureus *(*p *= 0.0319).

**Conclusions:**

Our findings suggest that HLA-DRB1*03 may be involved in susceptibility to nasal carriage of *S. aureus *in transplant patients. In addition, HLA-A*68 (dialysis patients) and HLA-A*03 and HLA-B*15 (transplant patients) appear to be associated with increased resistance to *S. aureus *nasal carriage.

## Background

*Staphylococcus aureus *is considered an important etiologic agent due to its high frequency and pathogenicity, causing diseases in both healthy and immunocompromised individuals. This microorganism is able to spread easily in the hospital environment and to develop resistance to antimicrobial agents [1,2].

Approximately 20% of adults are carriers of *S. aureus *in the nasopharynx, another 30% carry it intermittently, and 50% are not carriers [3,4]. Persistent nasal carriers of *S. aureus *are at higher risk of developing infection [5,6]. Dialysis and kidney transplant patients are particularly prone to infection due to constant exposure to risk factors and immunosuppression [2,7].

Infection is directly related to individual immunity and the major histocompatibility complex (MHC)--a set of genes found on the short arm of human chromosome 6--plays a crucial role in determining susceptibility to diseases. Specifically, MHC contains a group of genes that code for several proteins expressed on the surface of a variety of cell types. In humans, these molecules are known as human leukocyte antigens (HLA) [8].

Molecules of the HLA system are classified as class I (HLA-A, -B and -C), class II (HLA-DR, -DQ and -DP), and class III. These molecules are highly polymorphic glycoproteins that differentiate themselves from one another by their location in tissues and their function. Class I molecules are present on the surface of all nucleated cells and are responsible for presenting peptides to cytotoxic T cells (CD8^+^). Class II molecules are distributed less widely, being found primarily on the surface of cells linked directly to the immune response (such as macrophages, monocytes, dendritic cells, Langerhans cells, B lymphocytes, and activated T lymphocytes), with the purpose of presenting peptides to regulatory T cells (CD4^+^). The class III region does not encode histocompatibility molecules, but rather other molecules such as tumor necrosis factors, C4 and C2 proteins, complement factor B, heat shock protein, and 21-hydroxylase enzyme [9-12].

Different HLA specificities have been shown to be more frequent in individuals suffering from certain diseases, and characterizing these molecules may have important clinical implications [13-15].

The role of the HLA system in pathogenesis has been studied in several diseases, such as skin diseases [16-18], tuberculosis [19,20], psychiatric illnesses [21,22], as well as hearing [23,24], sight [25,26] and kidney [27] problems. Nasal carriage of *Staphylococcus spp*. has been investigated in dialysis and kidney transplant patients [28,29]; however, only a few studies have investigated the involvement of genetic factors in this carriage. A literature review returned only one study, published in 1983, investigating the association between HLA molecules and nasal carriage of *S. aureus *in healthy laboratory workers and outpatients [30].

Thus, this study aimed to investigate the association between HLA class I (HLA-A and -B) and class II (HLA-DRB1) molecules and nasal carriage of *S. aureus *in dialysis and kidney transplant patients at a hospital in Southern Brazil.

## Methods

The study population consisted of 70 renal patients undergoing dialysis and 46 kidney transplant patients, totaling 116 patients. All subjects were typed for HLA class I (A, B) and class II (DRB1) molecules and evaluated for nasal carriage. Biological material, nasal discharge and blood were collected between June and November 2009.

### *Staphylococcus aureus *identification

Nasal swab samples of *S. aureus *were isolated from the nasal cavity of patients undergoing dialysis or kidney transplantation by inserting sterile swabs into both nostrils. The material was placed in Petri dishes (90 × 15 mm) containing mannitol salt agar (MSA) (Becton Dickinson & Co., BD Diagnostic Systems, USA), transferred to a test tube containing trypticase soy broth (TSB) with 6.5% NaCl, and then placed in a stove for 24 hours. After 24-48 hours of incubation at 37°C, suspected *S. aureus *colonies were subjected to Gram staining and immersion microscopy. Those identified as Gram-positive cocci arranged in grape-like clusters were transferred to a TSB medium with 6.5% NaCl. The tube coagulase test was performed after six hours of incubation. After the identification tests, samples were stored in a TSB medium with glycerol (20%) and frozen at -20°C for later analysis.

### HLA typing

Human DNA extraction: Peripheral blood was collected by venipuncture in vacuum tubes containing EDTA anticoagulant, and genomic DNA was extracted using EZ-DNA reagent, according to the manufacturer's instructions (Biological Industries, Kibbutz Beit, Haemek, Israel).

Genetic polymorphism of HLA molecules: One Lambda LABType^® ^SSO kit was used in combination with the Luminex™ technology for typing of HLA class I (HLA-A and -B) and class II (HLA-DRB1) alleles.

### Statistical analysis

For data analysis, dialysis and transplant patients were subdivided into carriers and non-carriers of *S. aureus*. The number of times a given allele was found (n) and allele frequency (Af) were quantified in both patient groups. *P*-value was calculated using Fisher's exact test (*P*-value). Bonferroni correction (Pc-value) was used to calculate p values below 0.05. The odds ratio (OR) and confidence interval (95%CI) were calculated whenever the p value was lower than 0.05.

### Ethical aspects

The study was approved by the Research Ethics Committee of Universidade Estadual de Maringá (protocol no. 212/2009), and written informed consent was obtained from all participants. The study was conducted in accordance with the provisions of the Declaration of Helsinki.

## Results

Of 70 dialysis patients, 30 (42.8%) were female and 40 (57.1%) male. Age ranged from 22 to 85 years. Thirty-seven patients (52.8%) were nasal carriers of *S. aureus *and 33 (47.1%) were non-carriers.

Of 46 patients evaluated in the transplant group, 16 (34.7%) were female and 30 (65.2%) male. Age ranged from 18 to 64 years. Thirty-four patients (74%) were nasal carriers of *S. aureus *and 12 (26%) were non-carriers.

### Study association with dialysis patients and their status as nasal carriers or non-carriers of *Staphylococcus aureus*

Analysis of the frequency of class I, A and B alleles, and class II, DRB1 alleles, (Table 1) showed that HLA-A*02 was the most frequent allele in both carriers (25.5%) and non-carriers (21.2%). HLA-A*68 was not observed in the carrier group, but the allele was observed six times in the non-carrier group (*p *= 0.0097). This result suggests a tendency towards protection or resistance against nasal carriage of *S. aureus*. Regarding HLA-B and -DRB1, no allele was shown to be involved in protection against or susceptibility to carriage of *S. aureus*.

**Table 1:** Allele frequency (HLA-A, HLA-B, and HLA-DRB1) in the group of renal patients undergoing dialysis, nasal carriers and non-carriers of *Staphylococcus aureus*

	Carriers		Non-carriers							Carriers		Non-carriers					
											
Allele	n	*Af*%	n	*Af*%	*P*-value	Pc-value	OR	95%CI	Allele	n	*Af*%	n	*Af*%	*P*-value	Pc-value	OR	95%CI
**HLA-A**									**HLA-B**								
**01**	9	12.1	6	9.0	ns	-			**05**	0	-	1	1.5	ns	-		
**02**	19	25.6	14	21.2	ns	-			**07**	4	5.4	6	9.0	ns	-		
**03**	6	8.1	7	10.6	ns	-			**08**	4	5.4	5	7.5	ns	-		
**11**	6	8.1	4	6.0	ns	-			**13**	1	1.3	0	-	ns	-		
**23**	2	2.7	2	3.0	ns	-			**14**	0	-	2	3.0	ns	-		
**24**	9	12.1	10	15.1	ns	-			**15**	1	1.3	3	4.5	ns	-		
**25**	3	4.0	0	-	ns	-			**16**	0	-	1	1.5	ns	-		
**26**	2	2.7	5	7.5	ns	-			**18**	7	9.4	6	9.0	ns	-		
**29**	3	4.0	1	1.5	ns	-			**22**	1	1.3	0	-	ns	-		
**30**	5	6.7	5	7.5	ns	-			**27**	2	2.7	0	-	ns	-		
**31**	3	4.0	1	1.5	ns	-			**35**	6	8.1	4	6.0	ns	-		
**32**	2	2.7	2	3.0	ns	-			**38**	3	4.0	3	4.5	ns	-		
**33**	2	2.7	2	3.0	ns	-			**39**	3	4.0	4	6.0	ns	-		
**34**	2	2.7	1	1.5	ns	-			**40**	1	1.3	2	3.0	ns	-		
**68**	0	-	6	9.0	0.0097	0.1552	-	-	**41**	0	-	1	1.5	ns	-		
**74**	1	1.3	0	-	ns	-			**42**	2	2.7	1	1.5	ns	-		
**HLA-DRB1**									**44**	6	8.1	5	7.5	ns	-		
**01**	3	4.0	5	7.5	ns	-			**45**	2	2.7	0	-	ns	-		
**03**	1	1.3	2	3.0	ns	-			**49**	2	2.7	2	3.0	ns	-		
**04**	10	13.5	13	19.7	ns	-			**50**	1	1.3	3	4.5	ns	-		
**07**	8	10.8	6	9.0	ns	-			**51**	7	9.4	8	12.1	ns	-		
**08**	5	6.7	2	3.0	ns	-			**52**	2	2.7	2	3.0	ns	-		
**09**	1	1.3	0	-	ns	-			**53**	1	1.3	2	3.0	ns	-		
**10**	3	4.0	3	4.5	ns	-			**55**	4	5.4	0	-	ns	-		
**11**	17	22.9	11	16.6	ns	-			**57**	3	4.0	0	-	ns	-		
**13**	6	8.1	7	10.6	ns	-			**58**	1	1.3	2	3.0	ns	-		
**14**	5	6.7	1	1.5	ns	-			**60**	2	2.7	1	1.5	ns	-		
**15**	3	4.0	7	10.6	ns	-			**61**	2	2.7	0	-	ns	-		
**16**	5	6.7	6	9.0	ns	-			**62**	4	5.4	0	-	ns	-		
**17**	3	4.0	3	4.5	ns	-			**65**	1	1.3	2	3.0	ns	-		
**18**	1	1.3	0	-	ns	-			**81**	1	1.3	0	-	ns	-		
**51**	1	1.3	0	-	ns	-											
**52**	2	2.7	0	-	ns	-											

*N *number of times the allele was found; *Af *allele frequency; *P*-value = calculated by Fisher's exact test; Pc-value = Bonferroni-corrected P-value; *OR *odds ratio; 95%*CI *95% confidence interval; *ns *non-significant (*p *> 0.05)

### Study association with kidney transplant patients and their status as nasal carriers or non-carriers of *Staphylococcus aureus*

As shown in Table 2, HLA-A*03 was more frequent in the non-carrier group (20.83%) than in the carrier group (5.88%) (*p *= 0.0486), which suggests a tendency towards protection against carriage of *S. aureus*. HLA-B*15 also showed a trend towards protection against carriage (carriers: 5.88%; non-carriers: 25%) (*p *= 0.0179). Regarding class II alleles, DRB1*03 appeared to be related to susceptibility to carriage of *S. aureus *(*p *= 0.0319).

**Table 2:** Allele frequency (HLA-A, HLA-B, and HLA-DRB1) in the group kidney transplant patients, nasal carriers and non-carriers of *Staphylococcus aureus*

	Carriers		Non-carriers							Carriers		Non-carriers					
											
Allele	n	*Af*%	n	*Af*%	*P*-value	Pc-value	OR	95%CI	Allele	n	*Af*%	n	*Af*%	*P*-value	Pc-value	OR	95%CI
**HLA-A**									**HLA-B**								
**01**	9	13.24	2	8.33	ns	-			**07**	5	7.35	1	4.17	ns			
**02**	21	30.88	6	25.0	ns	-			**08**	6	8.82	0	-	ns			
**03**	4	5.88	5	20.83	**0.0486**	0.7776	0.2375	0.0579-0.9738	**12**	0	-	1	4.17	ns			
**11**	2	2.94	0	-	ns	-			**13**	2	2.94	1	0.417	ns			
**23**	1	1.47	1	4.17	ns	-			**14**	3	4.41	0	-	ns			
**24**	9	13.24	2	8.33	ns	-			**15**	4	5.88	6	25.0	**0.0179**	0.4833	0.1875	0.0477-0.7371
**26**	2	2.94	1	4.17	ns	-			**18**	2	2.94	1	4.17	ns			
**28**	0	-	1	4.17	ns	-			**27**	0	-	1	4.17	ns			
**29**	4	5.88	0	-	ns	-			**35**	8	11.76	2	8.33	ns			
**30**	6	8.82	1	4.17	ns	-			**37**	2	2.94	1	4.17	ns			
**31**	2	2.94	1	4.17	ns	-			**38**	1	1.47	0	-	ns			
**32**	1	1.47	1	4.17	ns	-			**39**	2	2.94	0	-	ns			
**33**	3	4.41	0	-	ns	-			**40**	1	1.47	3	12.50	ns			
**36**	1	1.47	0	-	ns	-			**41**	1	1.47	1	4.17	ns			
**66**	1	1.47	0	-	ns	-			**42**	1	1.47	0	-	ns			
**68**	2	2.94	3	12.50	ns	-			**44**	5	7.35	3	12.50	ns			
**HLA-DRB1**									**45**	2	2.94	0	-	ns			
**01**	3	4.41	0	-	ns	-			**47**	0	-	1	4.17	ns			
**03**	12	17.65	0	-	**0.0319**	0.4785			**49**	4	5.88	0	-	ns			
**04**	9	13.24	3	12.50	ns	-			**51**	6	8.82	1	4.17	ns			
**07**	3	4.41	4	16.67	ns	-			**52**	3	4.41	0	-	ns			
**08**	2	2.94	2	8.33	ns	-			**53**	1	1.47	0	-	ns			
**09**	3	4.41	1	4.17	ns	-			**55**	1	1.47	0	-	ns			
**10**	0	-	1	4.17	ns	-			**57**	4	5.88	0	-	ns			
**11**	13	19.12	6	25.0	ns	-			**58**	2	2.94	1	4.17	ns			
**12**	0	-	2	8.33	ns	-			**62**	1	1.47	0	-	ns			
**13**	6	8.82	1	4.17	ns	-			**70**	1	1.47	0	-	ns			
**14**	5	7.35	0	-	ns	-											
**15**	7	10.29	2	8.33	ns	-											
**16**	2	2.94	2	8.33	ns	-											
**17**	2	2.94	0	-	ns	-											
**18**	1	1.47	0	-	ns	-											

*N *number of times the allele was found; *Af *allele frequency; *P*-value = calculated by Fisher's exact test; Pc-value = Bonferroni-corrected *P*-value; *OR *odds ratio; 95%*CI *95% confidence interval; *ns *non-significant (*p *> 0.05)

## Discussion

The molecules of the HLA system, which are directly linked to the immune response, play a crucial role in the susceptibility to or protection against infectious diseases [31]. The determination of these molecules is often used in association studies with various illnesses, allowing a search for specific markers of susceptibility or resistance [32,33]. In our study, we demonstrated that certain HLA alleles could be involved in nasal carriage of *S. aureus *in dialysis and kidney transplant patients.

During the literature review, we found only one study on HLA and nasal carriage of *S. aureus *[30], which employed the serological method of HLA typing. That study analyzed healthy laboratory workers and patients attending an outpatient clinic in Dublin and Galway, Ireland. A positive association was found between HLA-DR3 antigen and carriage of *S. aureus*, making individuals more susceptible to carrying the bacterium. Class I (HLA-Bw35) and class II (HLA-DR2 and HLA-DR1) antigens were also shown to be involved in resistance against non-carriage of the bacterium.

The present study contributed to confirm the involvement of HLA-DRB1*03 (serological equivalent DR3) as a possible genetic marker for susceptibility to nasal carriage of *S. aureus*. It also contributed to demonstrate the involvement of HLA class I specificities (-A*03, -A*68, and -B*15) as protective factors against nasal carriage of *S. aureus *in renal patients.

The importance of identifying patients carrying *S. aureus*, in particular those with a multi-resistant profile, lies in the fact that this pathogen is linked to endogenous infections [34]. In that sense, it is worth noting that infectious diseases exert selective genetic pressure, and the genes involved in the immune response are the most numerous and polymorphic of the human genome, thereby indicating the evolutionary advantages of the immune response to a wide variety of pathogens. This is evidenced by the participation of HLA in the presentation of foreign peptides to immune system cells [35].

## Conclusions

Our findings suggest that HLA-DRB1*03 may be involved in susceptibility to nasal carriage of *S. aureus *in transplant patients. In addition, HLA-A*68 (dialysis patients) and HLA-A*03 and HLA-B*15 (transplant patients) appear to be associated with increased resistance to *S. aureus *nasal carriage.

Further studies are warranted to clarify the role of HLA as a genetic marker in resistance against or susceptibility to carriage of *S. aureus*. Establishing the role of host genetic factors should contribute to a better understanding of infectious conditions as well as to develop new therapies according to the progress in the field of pharmacogenetics.

## Availability of supporting data

The data set supporting the results of this article is available in the repository of Universidade Estadual de Maringá (UEM), at UEM Digital Library, under no. VTLS 000184756/www.bce.uem.br.

## Competing interests

The authors declare that they have no competing interests.

## Authors' contributions

SDB carried out HLA typing, drafted the manuscript and revised it critically for the intellectual content. RRS participated in the collection of biological material, processing and analysis of data. JB participated in the design of the study, helped to draft the manuscript and revised it critically for the intellectual content. WVSJ participated in the design of the study and performed the statistical analysis. LBG participated in the collection of biological material, processing and analysis of data, helped to draft the manuscript and revised it critically for the intellectual content. All authors read and approved the final manuscript.
